# Rapid Eye Movement Behavior Sleep Disorder as a Primary Manifestation of Wilson Disease: A Gateway to Early Intervention

**DOI:** 10.7759/cureus.29618

**Published:** 2022-09-26

**Authors:** Diana Voloshyna, Anan Bseiso, Tanveer Ahamad Shaik, Swastika Sedhai, Alaa Abdelsamad, Maryam H Khan, Qudsia I Sandhu, Faraz Saleem, Muhammad Abu Zar Ghaffari

**Affiliations:** 1 School of Medicine, University of Michigan, Ann Arbor, USA; 2 College of Medicine, Al-Quds University, Jerusalem, PSE; 3 Cardiovascular Medicine, University of Louisville School of Medicine, Louisville, USA; 4 Medicine, Kathmandu University, Kathmandu, NPL; 5 Geriatrics, Michigan State University, Michigan, USA; 6 Internal Medicine, Rawalpindi Medical University, Lahore, PAK; 7 Medicine, Ghazi Khan Medical College, Dera Ghazi Khan, PAK; 8 Internal Medicine, Akhtar Saeed Medical and Dental College, Lahore, PAK

**Keywords:** neurology, wilson disease neuropsychiatric manifestation, rem sleep behavior disorder, rbd, wilson disease

## Abstract

Wilson's disease (WD) is one of the most prevalent genetic conditions in the world. The average onset age ranges between 5 and 35 years. The prognosis tends to be worse if the diagnosis is delayed. Neurocognitive and psychological disorders are the most common extrahepatic manifestations of WD. Moreover, rapid eye movement (REM) sleep behavior disorder (RBD) is discovered to have a significant correlation with neurodegenerative disorders, particularly WD. Several synucleinopathies, including WD, have an early prodromal stage that manifests as RBD or sleep behavior disorder. We hereby present a case of a 14-year-old patient with borderline ceruloplasmin levels and REM sleep disorder as an early manifestation of WD. RBD may be considered one of the earliest manifestations of such disorders and a vital phase of the disease's onset, as the patient may be more responsive to treatment at this point.

## Introduction

The inheritance pattern of Wilson's disease (WD) is autosomal recessive. It is caused by a lack of copper-transporting ATPase, which can be induced by one of many mutations in the ATP7B gene on chromosome 13 (q14.3). These mutations result in the decreased copper-binding ability of ceruloplasmin, and the free copper accumulates in different parts of the body, particularly the liver, cornea, and basal ganglia [1]. One in 30,000 to 100,000 people are affected globally [2]. Symptoms might range from liver illness to neuropsychiatric symptoms. It is estimated that about 40% of patients may experience neurological abnormalities, the most frequent of which are mobility impairments. In addition, multiple sleep disorders (SDs), such as rapid eye movement sleep behavior disorder (RBD), have been identified [1]. It has been reported that 42% to 80% of patients with WD have sleep problems [2], including insomnia, daytime sleepiness, poor sleep quality, RBD, cataplexy-like episodes (CLEs), and sleep paralysis [3]. Our inadequate understanding of the natural history of WD is reflected in the wide range of onset ages and clinical presentations observed [4]. Therefore, evaluating WD in a patient with sleep difficulties should be standard practice.

## Case presentation

A 14-year-old student presented to the outpatient department of a tertiary care hospital with complaints of violent movements during sleep, and numerous bed falls over the past few months. He easily recalled having vivid and threatening dreams, typically revolving around themes of persecution, falling, and combat. He is an above-average student and has no history of psychiatric illness. He reported incidents of aggression and rage that were primarily related to his vivid dreams, as he only experienced them after awakening from nightmares in which strangers typically beat him. Furthermore, he occasionally breaks his bed as a result of these occurrences in his dreams.

On reviewing his past family history, his father had similar instances of nightmares and suffered from regular mood swings, which grew more intense throughout the course of his son's lifetime. However, his father died of acute liver failure two years ago. He has no siblings, and his mother is a teacher with no family history of serious illnesses. The patient was observed to have fine hand tremors after a thorough examination. A review of other systems, including the CNS, revealed no evidence of cognitive deterioration (MMSE 30). He was diagnosed with violent dream enactment behavior and told to keep a dream journal for two weeks. The Epworth Sleepiness Scale, the Mayo Sleep Questionnaire, and the Pittsburgh Sleep Quality Index were used to evaluate his sleep quality and all of these assessments pointed toward poor sleep quality. Daytime electroencephalograms (EEGs) were repeated and revealed no abnormalities.

Given his family history of liver failure, behavioral disorder, and the presentation of SD, WD was one of the top differentials. A comprehensive series of tests were ordered to exclude WD, including serum ceruloplasmin and copper levels. His liver function tests, alanine transaminase (ALT) (85) and aspartate transaminase (AST) (70), were elevated, ceruloplasmin was borderline at 17, serum copper was low normal, and urine copper was unremarkable. His liver copper quantitative assay revealed three times the limit of copper in the absence of cholestatic jaundice. Kayser-Fleischer's ring (KFR) could not be detected by ophthalmological evaluation. The patient received a score of 3 on the Leipzig scoring system for WD (ceruloplasmin levels 1, liver copper assessment 1, and neuropsychiatric manifestations 1 ) (Table 1). So PCR-based techniques (M-ARMS-PCR and T-ARMS-PCR) were recommended for preliminary screening of mutations in the ATP7B gene.

**Table 1:** Leipzig scoring system for Wilson's disease KF: Kayser-Fleischer; ULN: upper limit of normal. *Or typical abnormalities at brain magnetic resonance imaging. ^¶^If no quantitative liver copper is available.

Typical clinical symptoms and signs		Other tests	
KF rings		Liver copper (in the absence of cholestasis)	
Present	2	>5x ULN (>4 micromol/g)	2
Absent	0	0.8-4 micromol/g	1
Neurologic symptoms*		Normal (<0.8 micromol/g)	–1
Severe	2	Rhodanine-positive granules¶	1
Mild	1	Urinary copper (in the absence of acute hepatitis)	
Absent	0	Normal	0
Serum ceruloplasmin		1-2x ULN	1
Normal (>0.2 g/L)	0	>2x ULN	2
0.1-0.2 g/L	1	Normal, but >5x ULN after D-penicillamine	2
<0.1 g/L	2	Mutation analysis	
Coombs-negative hemolytic anemia		On both chromosomes detected	4
Present	1	On one chromosome detected	1
Absent	0	No mutations detected	0
Total score	Evaluation		
4 or more	Diagnosis established		
3	Diagnosis possible, more tests needed		
2 or less	Diagnosis very unlikely		

A psychiatric evaluation and an MRI scan of the brain were also advised, which was later found unremarkable. An in-depth treatment plan was discussed with the family after they had counseling. Initial therapy was started primarily based on the chelating agent D-penicillamine 500 mg/day. The patient was strongly advised to avoid copper-rich foods and beverages, particularly during the first year of treatment. A follow-up visit was suggested for the patient's continued care and D-penicillamine dose adjustment once they receive the genetic testing results.

## Discussion

At presentation, hepatic symptoms of WD can be quite diverse, ranging from mild to severe. It may range from acute hepatitis, jaundice, hepatomegaly, fatty liver, increased serum aminotransferases, isolated splenomegaly, and compensated or decompensated cirrhosis accompanied by fulminant hepatitis [5].

KFR and brain involvement are the two most common extrahepatic manifestations. They could be the only early symptoms of the disease. Approximately 18% to 68% of patients develop neurological problems [4]. All WD-related publications and literature highlight the classic psychiatric manifestations of the disease and their treatment. According to studies, up to 64.8% of patients initially presented with psychiatric symptoms (with or without hepatic or neurologic abnormalities), and up to 20% of patients had consulted a psychiatrist preceding the diagnosis of WD [6].

Some of the earliest symptoms of WD are sleep abnormalities, such as daytime drowsiness, insomnia, CLEs, restless legs syndrome, and RBD, all of which can be confirmed by recordings of the patient's sleep [4]. The high prevalence of SD in WD patients has been theorized to be due to abnormal copper buildup in sleep pathways in the brain, particularly in the brainstem, resulting in impaired amino butyric acid-mediated synaptic transmission and affecting monoaminergic neurons (so-called REM-off neurons). Recent research suggests that RBD may be an early indicator of extrapyramidal syndromes in Parkinson's disease and other neurodegenerative illnesses, similar to what has been observed in other SDs resulting from brainstem dysfunction [7]. Parasomnia known as RBD is characterized by complex and frequently aggressive motor activities that occur during REM sleep [8]. 

The paralysis of skeletal muscles during normal REM sleep prevents the occurrence of any movement. However, this phenomenon is compromised in patients with RBD, leading to a wide range of motor behaviors. Movements can range in complexity from simple ones like talking, yelling, and shaking limbs to more complicated ones like making gestures, hitting, or punching that appear to be aimed at a specific target. Oftentimes, medical treatment is needed as a patient can sustain an injury due to RBD [1]. The neurodegenerative illness commonly associated with alpha-synuclein accumulation is the primary reason for concern in people with RBD. Patients with RBD have an increased risk of developing Parkinson's disease, multiple system atrophy, or dementia with Lewy bodies; these diseases affect more than 80% of RBD patients (DLB). Having the ability to detect prodromal neurodegeneration prior to the onset of clinical symptoms has important implications given RBD's status as a reliable clinical predictor of the beginning of neurodegenerative disorders. RBD provides a distinctive chance to investigate the progression of a neurodegenerative condition from its prodromal phases. RBD is a common symptom of neurodegeneration in the early stages of alpha-synucleinopathies [9]. 

According to one systematic study and meta-analysis, patients with WD had a high prevalence of sleep disruptions. Among the patients with WD, 54.1% reported sleep abnormalities, presenting with a 7.7-fold higher odds of sleep disturbances compared to control patients. Our findings suggested that patients with WD have a higher prevalence of RBD and have more severe RBD symptoms in comparison with control patients [3].

Recognizing the symptoms that may indicate WD or knowing who in the family should be screened is the first step in making a diagnosis. A complete blood count, liver biochemistry tests, and serum ceruloplasmin level are the first steps in the diagnostic process. Next comes an eye exam using a slit-lamp or optical coherence tomography, and finally a 24-hour urine copper excretion sample (algorithm 1). Classic WD symptoms include low serum ceruloplasmin and the presence of KFRs in persons aged 5-35 years. However, only around half of the people with WD have these symptoms [10].

If the identified patient (proband) has been tested, and if both mutations have been detected then ATP7B mutation analysis should be the first step in family screening for siblings [11]. Patients with liver, neurological, or psychiatric problems who have no other known cause should be evaluated for WD, as should first-degree relatives of patients with WD. At a conference devoted to Wilson and Menkes' disease specialists in Leipzig, Germany, the criteria were developed by a panel of experts [11]. The later conference in Leipzig devised a scoring system to help evaluate whether or not to continue diagnostic testing for WD and the level of certainty associated with that diagnosis [12].

Early-stage diagnoses of WD are typically accompanied by only modest to moderate elevations in serum aminotransferases [13]. In most cases, the AST level is greater than the ALT level [11]. WD is always fatal if left untreated. Typically, development is slow and steady, although decline can come suddenly at any time. Liver failure is responsible for most deaths, with complications from the underlying progressive neurologic disease accounting for the rest [11]. Patients with advanced liver disease who actually start and continue treatment for WD have an excellent prognosis. Prognosis is good in patients without severe liver disease, while medication may increase neurologic symptoms in some patients, especially at the start of therapy [11]. After receiving treatment, the percentage of RBD prevalence in WD dropped from 11.2% to 6.7% [3]. In light of the above-mentioned literature, it can be established that RBD may present before any other WD symptoms. Thus, we propose that SD evaluations be conducted more frequently in WD, since an early diagnosis may aid in preventing further major psychological and neurological problems.

## Conclusions

The delay in diagnosis of WD has historically been described in the literature as the age of presentation and the primary features can vary widely. RBDs in young patients should be recognized and investigated. Furthermore, patients with a family history of the diagnosed or suspected cases should always be ruled out for WD. Early diagnosis and management can help reduce morbidity and mortality.

## References

[1] Limongi JC (2014). REM sleep behavior disorder, neurodegeneration and Wilson's disease. Arq Neuropsiquiatr.

[2] Poujois A, Woimant F (2019). Challenges in the diagnosis of Wilson disease. Ann Transl Med.

[3] Xu J, Deng Q, Qin Q, Vgontzas AN, Basta M, Xie C, Li Y (2020). Sleep disorders in Wilson disease: a systematic review and meta-analysis. J Clin Sleep Med.

[4] Tribl GG, Bor-Seng-Shu E, Trindade MC, Lucato LT, Teixeira MJ, Barbosa ER (2014). Wilson's disease presenting as rapid eye movement sleep behavior disorder: a possible window to early treatment. Arq Neuropsiquiatr.

[5] Boga S, Ala A, Schilsky ML (2017). Hepatic features of Wilson disease. Handb Clin Neurol.

[6] Zimbrean P, Seniów J (2017). Cognitive and psychiatric symptoms in Wilson disease. Handb Clin Neurol.

[7] Jernajczyk W, Litwin T, Członkowska A, Bembenek JP (2022). Sleep disturbances in newly diagnosed treatment-naïve patients with Wilson's disease. Acta Neurol Belg.

[8] Peever J, Luppi PH, Montplaisir J (2014). Breakdown in REM sleep circuitry underlies REM sleep behavior disorder. Trends Neurosci.

[9] Postuma RB, Gagnon JF, Montplaisir JY (2012). REM sleep behavior disorder: from dreams to neurodegeneration. Neurobiol Dis.

[10] Steindl P, Ferenci P, Dienes HP (1997). Wilson's disease in patients presenting with liver disease: a diagnostic challenge. Gastroenterology.

[11] (2022). Wilson Disease: Clinical Manifestations, Diagnosis, and Natural History. https://www.uptodate.com/contents/wilson-disease-clinical-manifestations-diagnosis-and-natural-history.

[12] Ferenci P, Caca K, Loudianos G (2003). Diagnosis and phenotypic classification of Wilson disease. Liver Int.

[13] Schilsky ML, Scheinberg IH, Sternlieb I (1994). Liver transplantation for Wilson's disease: indications and outcome. Hepatology.

